# Fear of Negative Evaluation Moderates the Effect of Subliminal Fear Priming on Rejection of Unfair Offers in the Ultimatum Game

**DOI:** 10.1038/srep31446

**Published:** 2016-08-23

**Authors:** Haruto Takagishi, Takayuki Fujii, Kuniyuki Nishina, Hiroyuki Okada

**Affiliations:** 1 Brain Science Institute, Tamagawa University, 6-1-1 Tamagawagakuen, Machida, Tokyo 194-8610, Japan; 2 Graduate School of Brain Sciences, Tamagawa University, 6-1-1 Tamagawagakuen, Machida, Tokyo 194-8610, Japan; 3 Department of Engineering, Tamagawa University, 6-1-1 Tamagawagakuen, Machida, Tokyo 194-8610, Japan

## Abstract

The purpose of this study was to examine whether the tendency to fear negative evaluation moderates the effect of fear emotion on the rejection of unfair offers in the ultimatum game (UG). A photograph of a fearful face or landscape was displayed subliminally (i.e., for 10 ms) before the proposer’s offer in the UG was presented to participants. We used the Fear of Negative Evaluation Scale (FNES) to measure participants’ anxiety regarding negative evaluations from others. Results showed a significant interaction between FNES and condition (fearful face vs. landscape) in relation to the rejection of an unfair offer. Furthermore, the mean rejection rate of an unfair offer was significantly higher in the fearful face condition relative to that in the landscape condition among participants whose FNES scores were higher than the median; however, this difference was not observed in participants whose FNES scores were lower than the median. These results suggest that fear of negative evaluation moderates the effect of subliminal fear priming on the rejection of unfair offers in the UG, and that negative emotion induced by unconscious stimuli enhances rejection of these unfair offers.

Factors leading to the rejection of unfair offers in the ultimatum game (UG)^1^ have attracted attention in various fields, such as psychology, economics, biology, and neuroscience, and a large number of studies on this topic have been conducted in recent years^2^,^3^,^4^,^5^. The UG is a simple two-person economic game in which one player (i.e., the proposer) decides how to divide a set amount of money between him or herself and a second player (i.e., the responder), and the responder decides whether to accept or reject the proposer’s offer. If the responder accepts the offer, the players receive amounts of money corresponding to the proposer’s offer; however, if the responder rejects the offer, neither player receives any money. If both players are motivated to maximize their own payoffs in the UG, the proposer should offer the minimum amount resources to the responder, and the responder should accept that offer; however, previous experimental studies have shown that almost all proposers offer fair proposals, and that responders typically reject unfair offers^6^. These results indicate that people are averse to unfairness^7^,^8^ and disprove economic models indicating that people are self-interested and motivated to maximize their own gain. Previous experimental studies conducted in various countries have examined participants’ aversion to unfairness using the UG^9^,^10^ and confirmed that the behavioural tendency to reject unfair offers was observed universally.

Why do people reject unfair offers in the UG? Using the proximate factor explanation, many studies have shown that rejection is driven by responders feeling negative emotions^4^,^11^,^12^,^13^,^14^,^15^, such as anger^12^,^13^ or disgust^4^,^11^. Although these negative emotional responses cause responders to abandon the opportunity to receive some money, and despite that the expression of negative emotions towards is often considered inappropriate behaviour in many societies^16^, some researchers have considered that the rejection of unfair offers is an adaptive reaction from an evolutionary perspective^3^,^5^,^17^,^18^,^19^. Burnham^17^ found that men with high testosterone levels showed a high rejection rate in response to unfair offers in the UG. Because testosterone level can indicate aggression and dominance^20^, Burham intepreted these results to mean that people who faced an unfair offer felt challenged by the proposer and rejected it to show their own dominance over others. Furthermore, Yamagishi *et al*.^5^ found a positive association between the tendency to assert one’s strength and rejection of unfair offers in the UG. Thus, the rejection of unfair offers in the UG may reflect the intention to avoid being subjugated by the proposer and signal that the unfairness is not acceptable^18^. Indeed, while rejection behaviour incurs a material cost in relation to short-term relationships with others, it also prevents future exploitation in long-term relationships^3^,^18^,^19^. Therefore, people tend to care about evaluations from others and to show their dominance to avoid incurring a negative evaluation. Thus, experiencing the fear emotion with regard to negative evaluation from others, as well as anger and disgust towards an unfair proposal, may play an important role in the decision to reject an unfair offer. Although one study reported that a sad mood induced the rejection of unfair offers in the UG^15^, to our knowledge, no prior study has focused on the role of fear emotion in this context. As previous studies have found that fear induces defensive aggression^21^ and because rejection of unfair offers in the UG is thought to reflect aggression^17^,^22^, it is likely that fear emotion will induce the rejection of unfair offers in the UG.

Fear of negative evaluation from others has been examined in research on social anxiety conducted in the fields of psychiatry or clinical psychology^23^. Social anxiety is characterised by the fear of negative evaluation from others, and many studies have shown that people with high social anxiety feel excessive fear and anxiety during or prior to public speaking^24^,^25^,^26^, and that their autonomic nervous system responses (e.g., heart rate and skin conductance response) increase significantly more than those of people with low social anxiety. Furthermore, in relation to facial recognition abilities, people with high social anxiety show overactivation of fear-related brain areas when they view a negative emotional expression on a face^27^,^28^,^29^. Interestingly, people with high social anxiety were found to show an autonomic nervous system response when a photograph of a fearful face was displayed in subliminal presentation^27^. In brief, people with high social anxiety are likely to both feel fear in social situations and be sensitive to social threats (e.g., negative evaluation from others and social exclusion), and this psychological mechanism might be processed unconsciously. Thus, fear emotion may enhance sensitivity to social threats and motivate people with high social anxiety to engage in appropriate behaviours in social situations in order to avoid negative evaluation from others. If the rejection of unfair offers in the UG is a defensive aggression tactic used to avoid negative evaluation from the proposer, then the tendency to fear negative evaluations might moderate the effect of fear emotion on offer rejection behaviour.

## The Current Study

In this study, we used subliminal fear priming to evoke participants’ fear emotion by displaying a photograph of a fearful face for a duration that was too short to reach conscious awareness, and then compared rejection rates in this condition with that in the condition in which a landscape photograph was displayed. Furthermore, we used the Fear of Negative Evaluation Scale^30^ (FNES) to measure participants’ fear of negative evaluation from others.

### Prediction

We predicted an interaction effect between FNES score and condition (fearful face vs. landscape) in relation to rejection of unfair offers in the UG, such that unfair offers would be more likely to be rejected in the fearful face condition relative to the landscape condition among people with a high FNES score.

## Results

The FNES demonstrated good reliability (Cronbach’s alpha = 0.91), and our participants’ mean score was 16.8 (*SD* = 7.4). FNES scores did not differ significantly between men (*M* = 16.7, *SD* = 6.4) and women (*M* = 16.9, *SD* = 8.3), *t* (68) = 0.11, *p* = 0.911.

To examine the effect of the target’s sex on rejection rates, we compared mean rejection rates of unfair offers (out of ¥1000) for female and male fearful faces for each proposer’s offer. No effect of target’s sex was found: ¥200/20% offer: female face: *M* = 0.49, *SD* = 0.50, male face: *M* = 0.60, *SD* = 0.49, *t* (69) = 1.73, *p* = 0.088; ¥100/10% offer: female face: *M* = 0.73, *SD* = 0.45, male face: *M* = 0.80, *SD* = 0.40, *t* (69) = 1.52, *p* = 0.133. Therefore, we combined the rejection rates for female and male fearful faces to form an overall rejection rate for the fearful face condition.

We compared unfair offer rejection rates between the first and final six of the 12 trials completed, to examine the effect exerted by the order of proposers’ offers. A two-way analysis of variance revealed no significant main effects of condition (*F* (1, 69) = 0.044, *p* = 0.835) or order (first six trials vs. final six trials; *F* (1, 69) = 1.363, *p* = 0.247), and the interaction between condition and order was nonsignificant (*F* (1, 69) = 0.840, *p* = 0.363). Therefore, we concluded that the order of the proposers’ offers did not affect unfair offer rejection rates.

Mean rejection rates are shown in Fig. S1. We conducted an analysis of covariance of rejection of unfair offers with the two within-subject factors of condition (fearful face and landscape) and level of offer (¥100/10% and ¥200/20%), and FNES score as a covariate. The effects of offer (*F* (1, 68) = 8.72, *p* = 0.004), condition (*F* (1, 68) = 4.86, *p* = 0.031), and condition × FNES score (*F* (1, 68) = 6.06, *p* = 0.016) were significant. However, the effects of FNES score (*F* (1, 68) = 0.001, *p* = 0.977), offer × FNES score (*F* (1, 68) = 0.68, *p* = 0.414), offer × condition (*F* (1, 68) = 0.38, *p* = 0.543), and offer × condition × FNES score (*F* (1, 68) = 0.38, *p* = 0.540) were nonsignificant.

As the interaction between condition and FNES score was significant, we combined rejection rates for the ¥100 (10%) and ¥200 (20%) offers and used this value as an indicator of the total mean rejection rate for unfair offers, before examining whether FNES scores differed according to the effect of subliminal fear priming on the rejection of unfair offers. A paired *t* test revealed that the mean rejection rate shown by participants whose total FNES scores were higher than the median (>17) in the fearful face condition (*M* = 0.68, *SD* = 0.35) was significantly higher relative to that of the landscape condition (*M* = 0.59, *SD* = 0.37), *t* (31) = 2.35, *p* = 0.025. However, this difference was not observed in participants whose total FNES scores were lower than the median (<17) (fearful face condition: *M* = 0.61, *SD* = 0.33; landscape condition: *M* = 0.66, *SD* = 0.34; *t* (32) = 1.31, *p* = 0.198).

**Figure 1:**
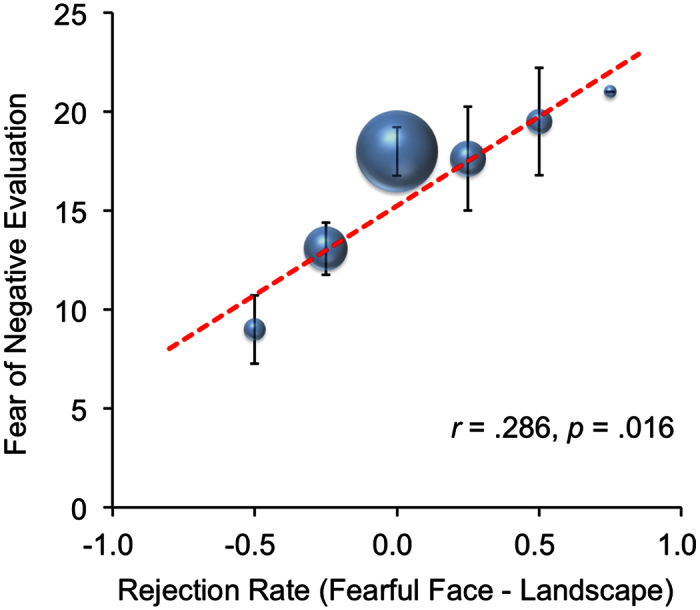
Relationship between rejection rate (fearful face condition minus landscape condition) and total Fear of Negative Evaluation Scale score. Error bars indicate standard errors and the size of the bulb indicates the size of the sample.

We then performed an additional analysis using the mean rejection rate for the fearful face condition minus that for the landscape condition as an indicator of the effect of subliminal fear priming on the rejection of unfair offers. Total FNES scores were positively correlated with the effect of subliminal fear priming on the rejection of unfair offers (*r* = 0.286, *p* = 0.016; Fig. 1).

## Discussion

While a previous study found that a sad mood induced rejection of unfair offers in the UG^15^, no prior studies have examined whether fear emotion plays a role in inducing rejection of these offers. In the current study, an interaction effect of FNES and condition in terms of the rejection of unfair offers in the UG was observed, such that the rejection rate for unfair offers was only higher in the fearful face condition, relative to that in the landscape condition, for participants with a high level of fear of negative evaluation. Moreover, total FNES scores were positively correlated with the difference in rejection rates between the fearful face and landscape conditions. The higher the FNES score, the more effect the presentation of a fearful face had on rejection in the UG. These results indicate that fear of negative evaluation moderated the effect of subliminal fear priming on the rejection of unfair offers in the UG, and that fear emotion, as induced by unconsciously perceived stimuli, promoted the rejection of unfair offers. This is the first study to show the effect of fear expression in rejection of unfair offer in the UG and show the effect of subliminal priming on the rejection of unfair offers in the UG. Our results suggest that this rejection tendency is an automatic and unconscious process.

Subliminal fear priming induced the rejection of unfair offers in the UG only in people with a high level of fear of negative evaluation, which implies that fear priming alone does not affect the rejection of unfair offers. The results of prior research in cognitive neuroscience might explain this finding. Recently, researchers’ attention has been focused on the role of the amygdala, i.e., the nucleus located in the limbic area, which is involved in the processing of learning, memory, and negative emotions^31^, in responses to unfair offers in the UG^32^,^33^. The amygdala responds to fearful or untrustworthy expressions on other people’s faces^34^,^35^, even when these stimuli are displayed for durations that are too short to reach conscious awareness^36^. Moreover, causality of the rejection of unfair offers and the function of the amygdala has been revealed^32^; thus, the amygdala plays an important role in warning against social threats, such as acquiring negative reputation. According to research on social anxiety, people who have high social anxiety show overactivation of the amygdala when faced with evaluation from others^25^,^26^,^37^ and when watching fearful face^27^,^28^,^29^. If the function of the amygdala and responsiveness to fearful faces is significantly higher in people who have high, vs. low, social anxiety, then the results observed in the current study can be explained by the function of the amygdala. However, the current study did not examine activity in the amygdala in response to subliminal fear priming; therefore, further research is required to determine if the activation of the amygdala induced by subliminal fear priming is associated with rejection rates in the UG.

The results of this study are consistent with the theoretical model of rejection of unfair offers in the UG, which has been suggested to reflect a tendency to avoid negative reputation^5^,^18^ and to result in long-term benefits, such as avoiding future exploitation in social exchange settings^3^,^19^. That is, participants with high fear of negative evaluation scores might exhibit increased concern about their own reputations upon viewing any human face, resulting in their rejecting an unfair offer to avoid being considered timid. If this explanation is accurate, any photographs of human faces should also induce rejection of unfair offers in participants with high social anxiety. Further research is required to compare the effect of fearful and neutral faces in terms of the rejection of unfair offers in the UG.

We found an effect of subliminal fear priming on rejection of unfair offers in the UG only among people with high FNES scores. Will this association also be observed in the real life, beyond the laboratory? In other words, do people with high fear of negative evaluation who are treated unfairly by others in their daily lives feel fear emotion and retaliate against them? We suspect the answer to this question is “yes”. Unlike the laboratory, which lacks of a specific social context, people build a long-term relationship and share a variety of experiences with others in real life. According to evolutionary theory^3^,^19^, people reject unfair offers to avoid incurring negative evaluations and future exploitation from others. Because the reputation plays an important role for the maintenance of good relationship with others, people with high fear of negative evaluation may retaliate against others who treat them unfairly in their daily lives. However, some motivations arising from specific relationships (e.g., disrupting relationships and avoiding retaliations) and situations (e.g., status differences and power relations) might inhibit retaliation to others. Further research is needed to examine whether the results of this study are still observed in real life situations.

In order to examine this issue, the dairy diary method^38^ might be the best way. In this method, participants describe the daily events and their feelings about them every day over an extended period of time. This method allow for the examination of the association between life events and emotion experiences. By comparing the frequncy and the association bewteen experiences of fear and negative reputation-threatening events between people with high and low fear of negative evalution, we would be able to examine the above question.

## Methods

### Participants

Participants were 70 undergraduate students (35 women, 35 men; mean age = 19.7 years, *SD* = 1.3), who were recruited via posters distributed in classrooms; the monetary reward was emphasized at the point of recruitment. The methods were carried out in accordance with the approved guidelines. All experimental protocols were approved by the ethics committee at Tamagawa University and written informed consent was obtained from all participants.

### Apparatus

All participants played the UG alone in a quiet room, and their anonymity was protected (Fig. S2). We used a tachistoscope (IWATSU Electric Co., Ltd., IS-703) and a 22-inch flat screen CRT display (MITSUBISHI, RDF22IH) to display the subliminal stimuli. The tachistoscope is widely used in psychological experiments examining vision and can display stimuli for periods as short as 10 ms. Subliminal fear priming with photographs of fearful faces has been used by many previous researchers in the fields of cognitive neuroscience and psychology, and found to strongly induce the emotion of fear in participants^39^,^40^,^41^.

### Ultimatum Game

All participants played a one-shot UG 12 times in the role of responder, with their opponent changing with each new trial (Fig. S3). The proposer decided how to allocate ¥1,000 between him or herself and the responder, who then decided whether to accept or reject the proposer’s offer. If the responder accepted the offer, both players received the amount of money corresponding to that in the proposer’s offer; however, if the responder rejected the offer, neither player received any money. Although all participants were informed that their opponents were other students participating in the study, the offer amounts were determined by the experimenter in advance and randomly displayed to the responders; ¥500, ¥200, and ¥100 were each offered to the responder four times. Because people frequently reject offers below 20%^6^, we used ¥200 and ¥100 offer as the unfair offers. In addition, a photograph of a male or female fearful face was displayed immediately prior to presentation of the proposer’s offer in half of the trials, and a photograph of a landscape was displayed in the other half of the trials. Male and female fearful faces were each displayed three times: once for each offer (¥500, ¥200, and ¥100), and a photograph of a landscape was displayed in six times: twice for each offer (¥500, ¥200, and ¥100). The proportion of proposer’s offer is summarized in Table 1.

**Figure 2:**
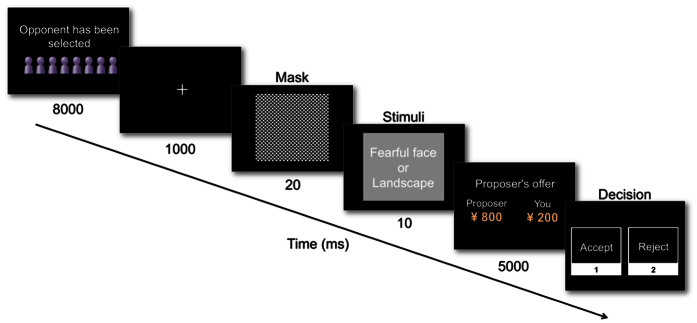
Timeline for each trial in the ultimatum game. In the proposer’s decision phase, the mask and target images (photographs of a fearful face or landscape) were displayed immediately prior to presentation of the proposer’s offer. In the responder’s decision phase, participants decided whether to accept or reject the offer by pressing a button.

The timeline for each trial in the UG is shown in Fig. 2. At the beginning of each trial, participants waited to be matched with a new opponent (8,000 ms); thereafter, a fixation point was displayed for 1,000 ms. We used forward masking to interfere with the perception of target stimuli (20 ms), before the image of a fearful face or landscape was displayed for 10 ms, followed by the proposer’s offer (5,000 ms). Participants (i.e., responders) then decided whether to accept or reject the proposer’s offer. At the end of the UG, participants’ rewards were determined based on the actual decisions made in two of their trials, which were chosen at random. The mean reward amount was ¥631.4 (*SD* = 185.4).

### Fear of Negative Evaluation

Subsequent to playing the UG, participants completed the FNES, which is a self-report scale that measures the tendency towards fear of social evaluation, and is used to assess social anxiety disorder in adults. The FNES contains 30 items, to which participants are required to respond ‘yes’ or ‘no’.

## Additional Information

**How to cite this article**: Takagishi, H. *et al*. Fear of Negative Evaluation Moderates the Effect of Subliminal Fear Priming on Rejection of Unfair Offers in the Ultimatum Game. *Sci. Rep.* **6**, 31446; doi: 10.1038/srep31446 (2016).

## Supplementary Material

Supplementary Information

## Figures and Tables

**Table 1:** Frequency and proportion of proposer’s offer in each condition.

	Proposer’s offer (Proposer : Responder)		
	500:500	800:200	900:100
Fearful face (Male)	1 (8.3%)	1 (8.3%)	1 (8.3%)
Fearful face (Female)	1 (8.3%)	1 (8.3%)	1 (8.3%)
Landscape	2 (16.6%)	2 (16.6%)	2 (16.6%)

## References

[b1] Güth W., Schmittberger R. & Schwarze B. An experimental analysis of ultimatum bargaining. J. Econ. Behav. Organ. 3, 367–388 (1982).

[b2] Hoffman E., McCabe K., Shachat K. & Smith V. Preferences, property rights, and anonymity in bargaining games. Game. Econ. Behav. 7, 346–380 (1994).

[b3] Nowak M. A., Page K. M. & Sigmund K. Fairness versus reason in the ultimatum game. Science 289, 1773–1775 (2000). 10976075 10.1126/science.289.5485.1773

[b4] Sanfey A. G., Rilling J. K., Aronson J. A., Nystrom L. E. & Cohen J. D. The neural basis of economic decision-making in the ultimatum game. Science 300, 1755–1758 (2003). 12805551 10.1126/science.1082976

[b5] Yamagishi T. . Rejection of unfair offers in the ultimatum game is no evidence of strong reciprocity. Proc. Natl. Acad. Sci. USA 109, 20364–20368 (2012). 23188801 10.1073/pnas.1212126109 PMC3528519

[b6] Camerer C. F. Behavioral game theory: Experiments in strategic interaction . Princeton, NJ: Princeton University Press (2003).

[b7] Fehr E. & Schmidt K. M. A theory of fairness, competition, and cooperation. Q. J. Econ. 114, 817–868 (1999).

[b8] Falk A. & Fischbacher U. A theory of reciprocity. Game. Econ. Behav. 54, 293–315 (2006).

[b9] Roth A. E., Prasnikar V., Okuno-Fujiwara M. & Zamir S. Bargaining and market behavior in Jerusalem, Ljubljana, Pittsburgh, and Tokyo: An experimental study. Am. Econ. Rev . 81, 1068–1095 (1991).

[b10] Henrich J. . ‘Economic man’ in cross-cultural perspective: Behavioral experiments in 15 small-scale societies. Behav. Brain Sci. 28, 795–815 (2005). 16372952 10.1017/S0140525X05000142

[b11] Chapman H. A., Kim D. A., Susskind J. M. & Anderson A. K. In bad taste: Evidence for the oral origins of moral disgust. Science 323, 1222–1226 (2009). 19251631 10.1126/science.1165565

[b12] Pillutla M. M. & Murnighan J. K. Unfairness, anger, and spite: Emotional rejections of ultimatum offers. Organ. Behav. Hum. Dec . 68, 208–224 (1996).

[b13] Xiao E. & Houser D. Emotion expression in human punishment behavior. Proc. Natl. Acad. Sci. USA 102, 7398–7401 (2005). 15878990 10.1073/pnas.0502399102 PMC1129129

[b14] Takagishi H., Fujii T., Kameshima S., Koizumi M. & Takahashi T. Salivary alpha-amylase levels and rejection of unfair offers in the ultimatum game. Neuroendocrinol. Lett. 30, 643–646 (2009). 20035253

[b15] Harlé K. M. & Sanfey A. G. Incidental sadness biases social economic decisions in the ultimatum game. Emotion 7, 876–881 (2007). 18039057 10.1037/1528-3542.7.4.876

[b16] Matsumoto D., Yoo S. H. & Fontaine J. Mapping expressive differences around the world the relationship between emotional display rules and individualism versus collectivism. J. Cross-Cult. Psychol. 39, 55–74 (2008).

[b17] Burnham T. C. High-testosterone men reject low ultimatum game offers. Proc. Biol. Sci . 274, 2327–2330 (2007). 17613451 10.1098/rspb.2007.0546 PMC1950304

[b18] Yamagishi T. . The private rejection of unfair offers and emotional commitment. Proc. Natl. Acad. Sci. USA 106, 11520–11523 (2009). 19564602 10.1073/pnas.0900636106 PMC2703666

[b19] Frank R. H. Passions within reason: The strategic role of the emotions . New York, NY: Norton (1998).

[b20] Mazur A. & Booth A. Testosterone and dominance in men. Behav. Brain Sci. 21, 353–363 (1998). 10097017

[b21] Simunovic D., Mifune N. & Yamagishi T. Preemptive strike: An experimental study of fear-based aggression. J. Exp. Soc. Psychol. 49, 1120–1123 (2013).

[b22] Crockett M. J., Clark L., Tabibnia G., Lieberman M. D. & Robbins T. W. Serotonin modulates behavioral reactions to unfairness. Science 320, 1739–1739 (2008). 18535210 10.1126/science.1155577 PMC2504725

[b23] Stein M. B. & Stein D. J. Social anxiety disorder. Lancet 371, 1115–1125 (2008). 18374843 10.1016/S0140-6736(08)60488-2

[b24] Etkin A. & Wager T. D. Functional neuroimaging of anxiety: A meta-analysis of emotional processing in PTSD, social anxiety disorder, and specific phobia. Am. J. Psychiatry 164, 1476–1488 (2007). 17898336 10.1176/appi.ajp.2007.07030504 PMC3318959

[b25] Lorberbaum J. P. . Neural correlates of speech anticipatory anxiety in generalized social phobia. Neuroreport 15, 2701–2705 (2004). 15597038

[b26] Tillfors M. . Cerebral blood flow in subjects with social phobia during stressful speaking tasks: A PET study. Am. J. Psychiatry 158, 1220–1226 (2001). 11481154 10.1176/appi.ajp.158.8.1220

[b27] Tsunoda T. . Social anxiety predicts unconsciously provoked emotional responses to facial expression. *Physiol. Behav*. 93, 172–176 (2008). 17889911 10.1016/j.physbeh.2007.08.012

[b28] Phan K. L., Fitzgerald D. A., Nathan P. J. & Tancer M. E. Association between amygdala hyperactivity to harsh faces and severity of social anxiety in generalized social phobia. Biol. Psychiatry 59, 424–429 (2006). 16256956 10.1016/j.biopsych.2005.08.012

[b29] Stein M. B., Goldin P. R., Sareen J., Eyler Zorrilla L. T. & Brown G. G. Increased amygdala activation to angry and contemptuous faces in generalized social phobia. Arch. Gen. Psychiatry 59, 1027–1034 (2002). 12418936 10.1001/archpsyc.59.11.1027

[b30] Watson D. & Friend R. Measurement of social-evaluation anxiety. J. Consult. Clin. Psychol. 33, 448–457 (1969). 5810590 10.1037/h0027806

[b31] Baxter M. G. & Murray E. A. The amygdala and reward. Nat. Rev. Neurosci. 3, 563–573 (2002). 12094212 10.1038/nrn875

[b32] Gospic K. . Limbic justice—Amygdala involvement in immediate rejection in the ultimatum game. PLoS Biol . 9, e1001054 (2011). 21559322 10.1371/journal.pbio.1001054 PMC3086869

[b33] Haruno M., Kimura M. & Frith C. D. Activity in the nucleus accumbens and amygdala underlies individual differences in prosocial and individualistic economic choices. J. Cogn. Neurosci . 26, 1861–1870 (2014). 24564471 10.1162/jocn_a_00589

[b34] Adolphs R., Tranel D. & Damasio A. R. The human amygdala in social judgment. Nature 393, 470–474 (1998). 9624002 10.1038/30982

[b35] Morris J. S. . A differential neural response in the human amygdala to fearful and happy facial expressions. Nature 383, 812–815 (1996). 8893004 10.1038/383812a0

[b36] Freeman J. B., Stolier R. M., Ingbretsen Z. A. & Hehman E. A. Amygdala responsivity to high-level social information from unseen faces. J. Neurosci. 34, 10573–10581 (2014). 25100591 10.1523/JNEUROSCI.5063-13.2014 PMC6802589

[b37] Tillfors M., Furmark T., Marteinsdottir I. & Fredrikson M. Cerebral blood flow during anticipation of public speaking in social phobia: A PET study. Biol. Psychiatry 52, 1113–1119 (2002). 12460694 10.1016/s0006-3223(02)01396-3

[b38] Nezlek J. B. Diary Methods for Social and Personality Psychology. London: Sage (2012).

[b39] Sato W., Kubota Y. & Toichi M. Enhanced subliminal emotional responses to dynamic facial expressions. Front. Psychol . 5, 994–9994 (2014). 25250001 10.3389/fpsyg.2014.00994 PMC4158748

[b40] Brooks S. J. . Exposure to subliminal arousing stimuli induces robust activation in the amygdala, hippocampus, anterior cingulate, insular cortex and primary visual cortex: A systematic meta-analysis of fMRI studies. NeuroImage 59, 2962–2973 (2012). 22001789 10.1016/j.neuroimage.2011.09.077

[b41] Sweeny T. D., Grabowecky M., Suzuki S. & Paller K. A. Long-lasting effects of subliminal affective priming from facial expressions. Conscious. Cogn. 18, 929–938 (2009). 19695907 10.1016/j.concog.2009.07.011 PMC2784103

