# Supplementary material for: Fear of Negative Evaluation Moderates the Effect of Subliminal Fear Priming on Rejection of Unfair Offers in the Ultimatum Game
Source: Sci Rep. 2016 Aug 23;6:31446. doi: 10.1038/srep31446 (PMC4993993; doi:10.1038/srep31446)
Supplement: Supplementary Information [file srep31446-s1.doc]

**Supplementary Information**

Fear of Negative Evaluation Moderates the Effect of Subliminal Fear Priming on Rejection of Unfair Offers in the Ultimatum Game

Haruto Takagishi, Takayuki Fujii, Kuniyuki Nishina, Hiroyuki Okada


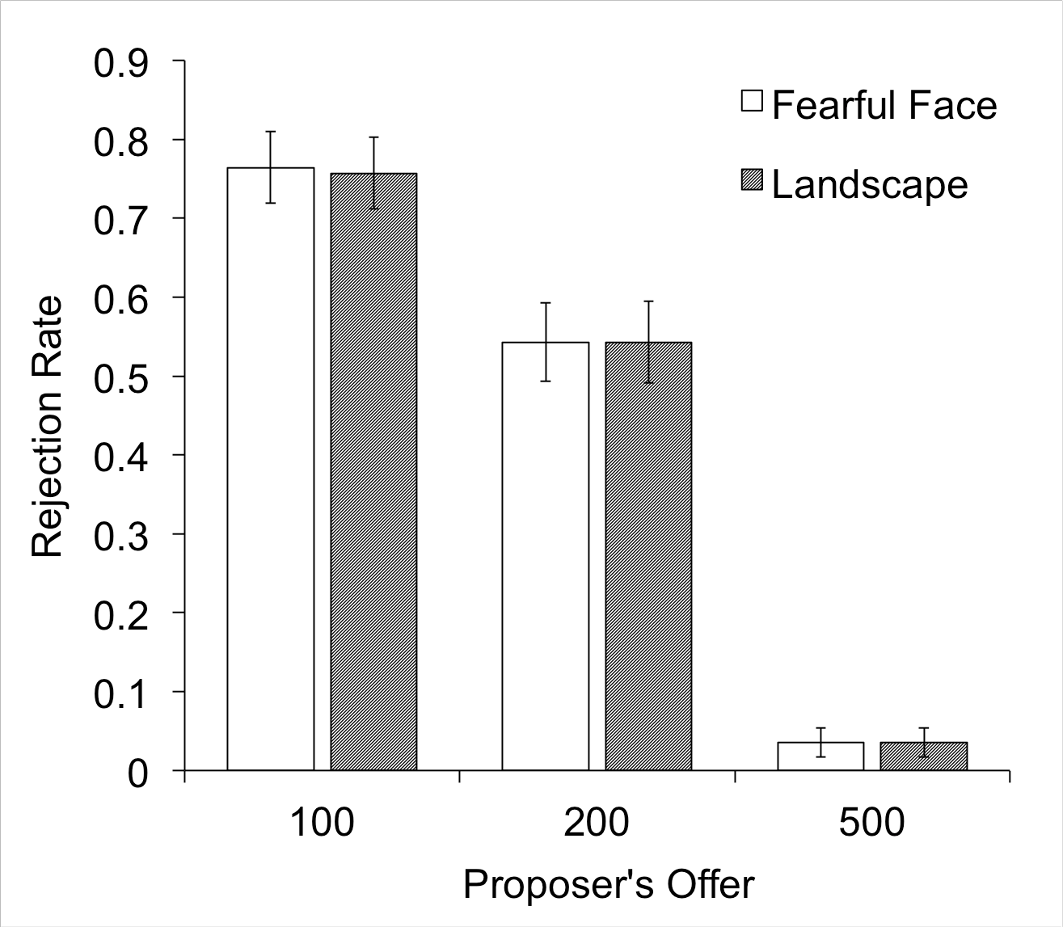


Fig. S1 Mean rejection rates in each offer. Error bars indicate standard error.


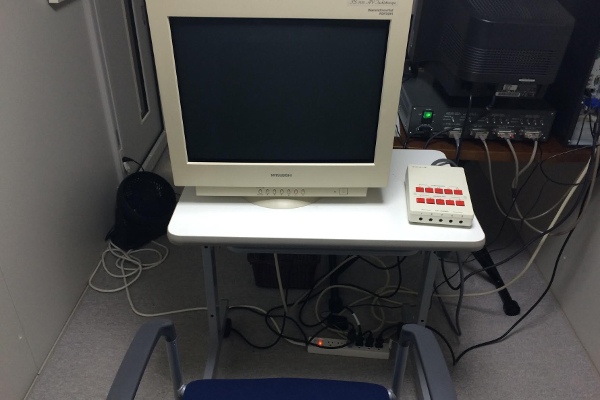

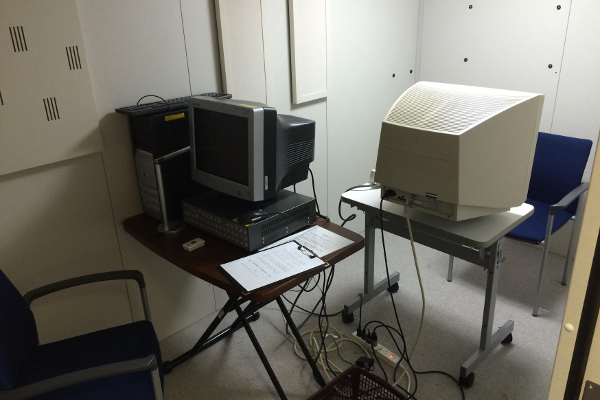


Fig. S2. Experimental setup. While the participants were playing the ultimatum game, the experimental staffs were not in the room.

a) Original Japanese Instructions


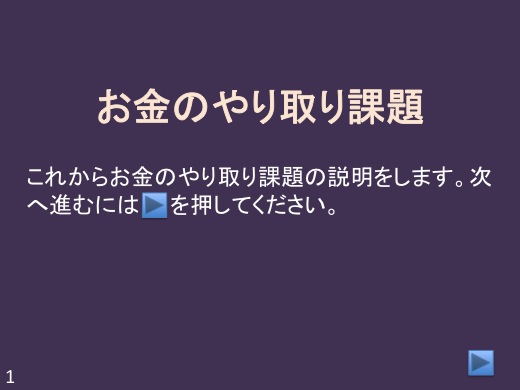

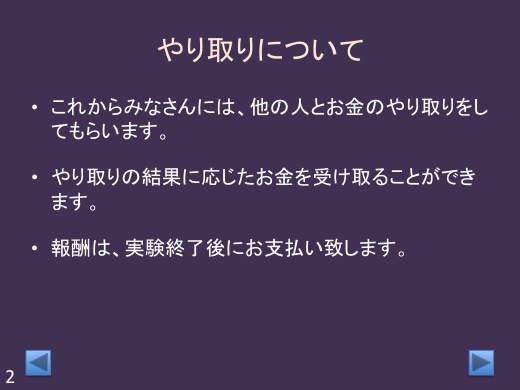

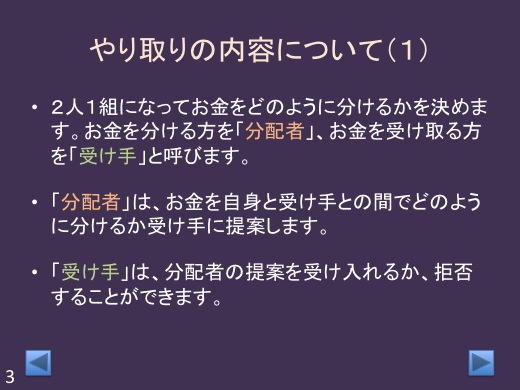

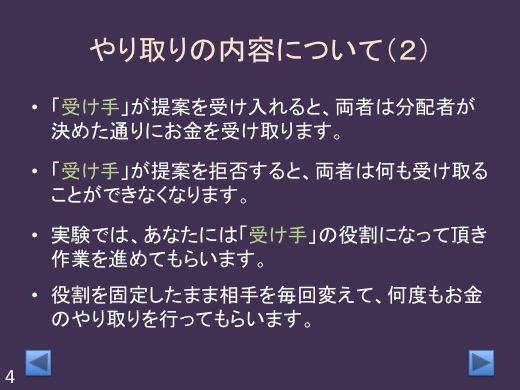

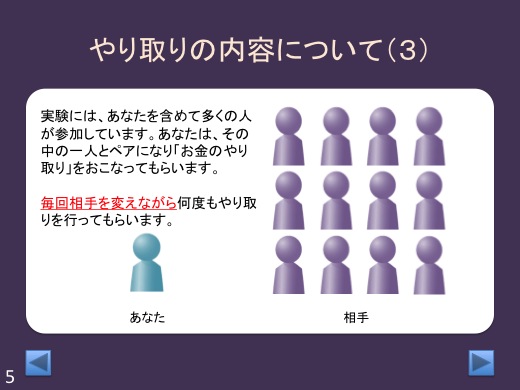

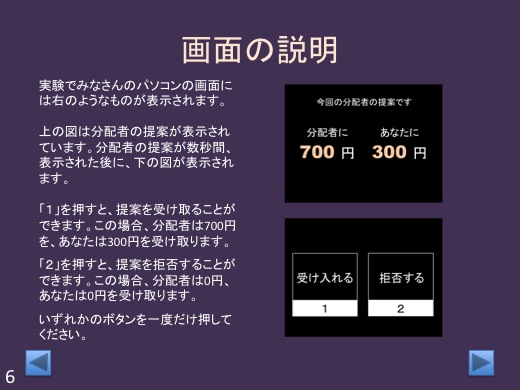

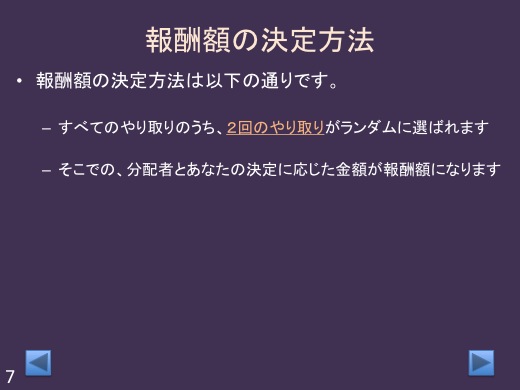

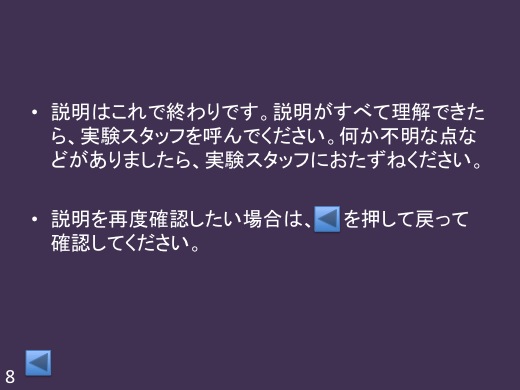


b) English Translation of Instructions


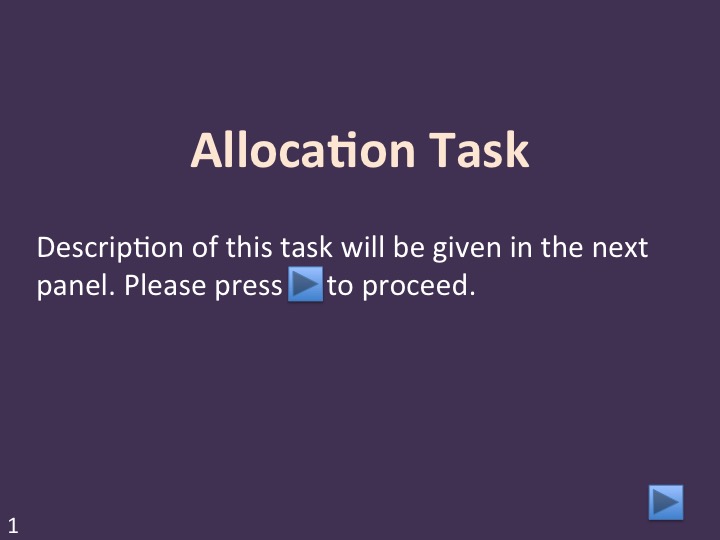

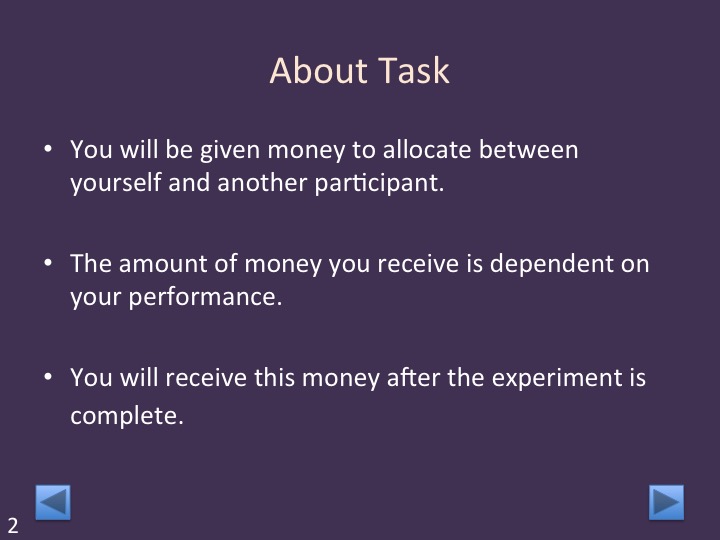

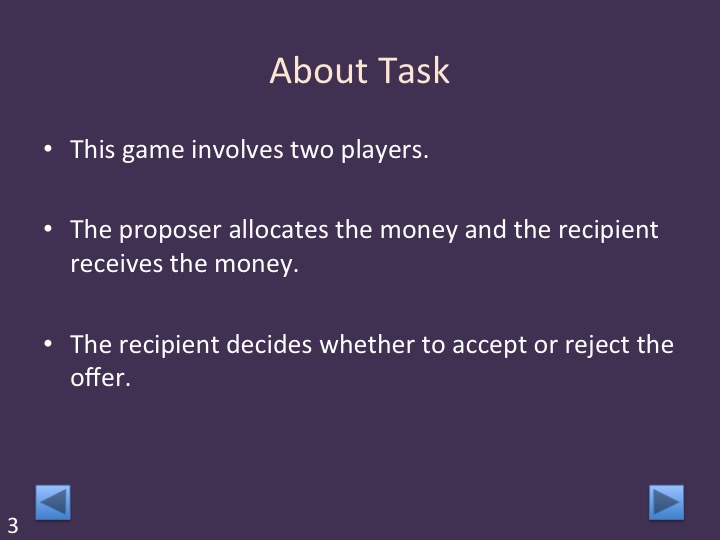

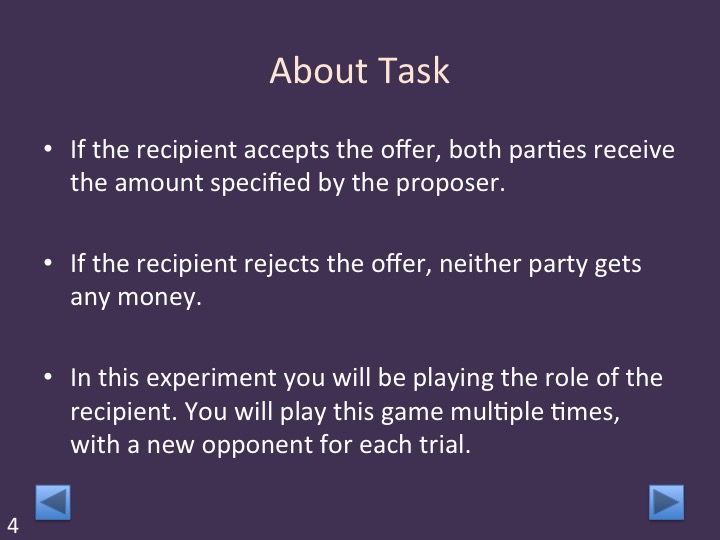

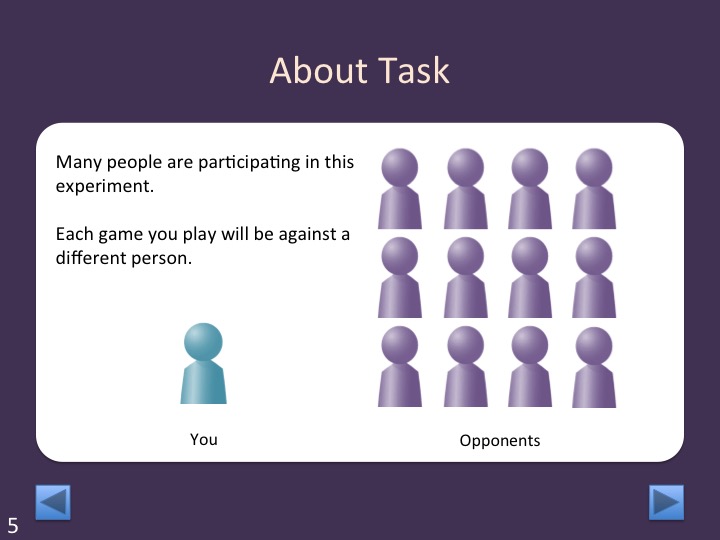

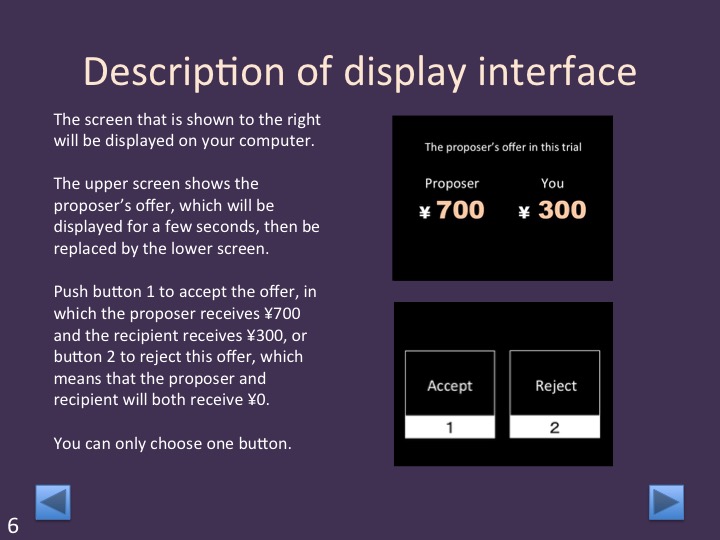

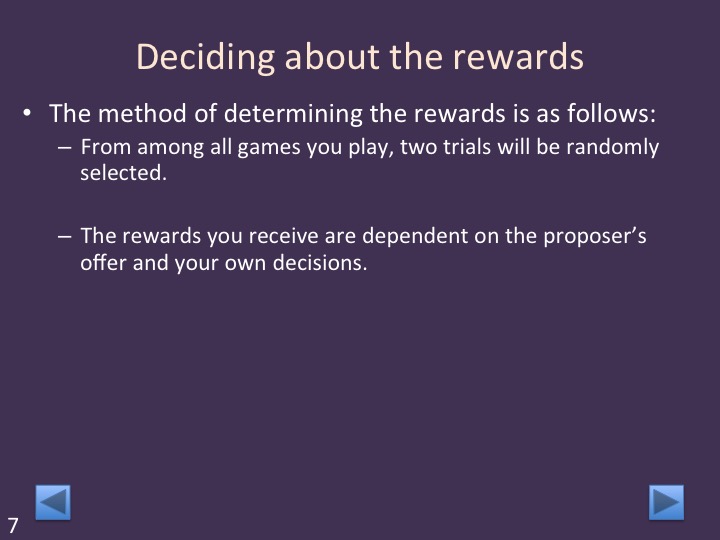

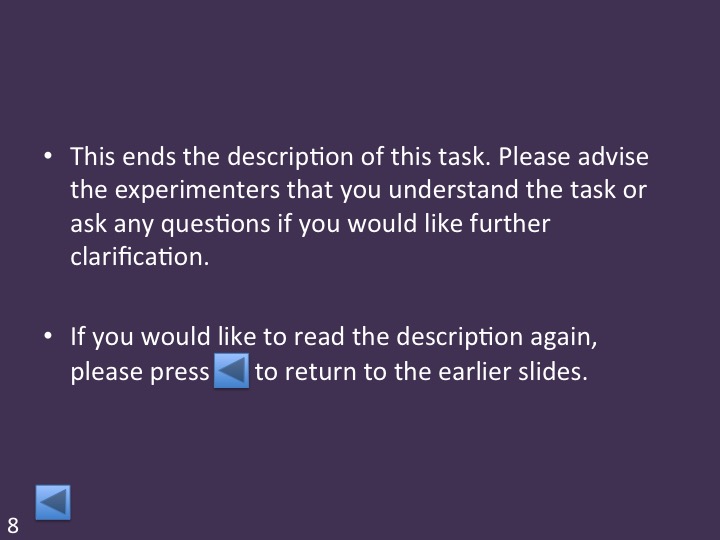


Fig S3. Instructions for the ultimatum game

Behavioural Data

| Sex | Age | Rejection rate | | | | | |  |  | |  | FNES score |
| --- | --- | --- | --- | --- | --- | --- | --- | --- | --- | --- | --- | --- |
|  |  | Fearful face  condition | | | Landscape  condition | | |  | First Trial | |  |  |
|  |  | ¥100 | ¥200 | ¥500 | ¥100 | ¥200 | ¥500 |  | Condition | Offer |  |  |
| F | 21 | 0.5 | 0 | 0 | 1 | 0 | 0 |  | Landscape | 200 |  | 7 |
| F | 19 | 1 | 0 | 0 | 0.5 | 0.5 | 0 |  | Landscape | 200 |  | 20 |
| M | 20 | 0 | 0.5 | 0 | 1 | 0.5 | 0 |  | Fearful face | 100 |  | 9 |
| M | 19 | 1 | 0 | 0 | 1 | 0 | 0 |  | Fearful face | 100 |  | 10 |
| F | 20 | 0 | 0 | 0 | 0 | 0 | 0 |  | Fearful face | 100 |  | 13 |
| M | 21 | 1 | 0 | 0 | 0.5 | 0 | 0 |  | Fearful face | 100 |  | 11 |
| F | 20 | 0 | 1 | 0 | 1 | 0 | 0 |  | Fearful face | 500 |  | 26 |
| M | 20 | 0.5 | 1 | 0 | 1 | 0.5 | 0 |  | Landscape | 500 |  | 17 |
| F | 20 | 0.5 | 0.5 | 0 | 0 | 0 | 0 |  | Landscape | 200 |  | 27 |
| M | 19 | 0.5 | 1 | 0 | 1 | 1 | 0 |  | Landscape | 500 |  | 15 |
| F | 19 | 1 | 0.5 | 0 | 1 | 1 | 0 |  | Fearful face | 200 |  | 12 |
| F | 21 | 0 | 0.5 | 0 | 0.5 | 0.5 | 0 |  | Fearful face | 100 |  | 10 |
| M | 20 | 0.5 | 0.5 | 0 | 0.5 | 0.5 | 0 |  | Landscape | 500 |  | 25 |
| M | 21 | 1 | 0 | 0 | 1 | 1 | 0 |  | Landscape | 200 |  | 12 |
| M | 20 | 0.5 | 0 | 0 | 0.5 | 0 | 0 |  | Landscape | 100 |  | 24 |
| M | 20 | 0.5 | 0 | 0 | 0.5 | 0 | 0 |  | Fearful face | 200 |  | 14 |
| F | 22 | 1 | 0 | 0 | 1 | 0 | 0 |  | Landscape | 100 |  | 24 |
| F | 21 | 0.5 | 0 | 0 | 0 | 0 | 0 |  | Landscape | 500 |  | 8 |
| F | 20 | 0.5 | 0 | 0 | 0.5 | 0 | 0 |  | Landscape | 200 |  | 18 |
| M | 19 | 1 | 0.5 | 0 | 1 | 1 | 0 |  | Landscape | 100 |  | 11 |
| M | 22 | 0 | 0 | 0 | 0 | 0 | 0 |  | Fearful face | 100 |  | 18 |
| F | 22 | 1 | 0.5 | 0 | 1 | 0 | 0 |  | Fearful face | 100 |  | 14 |
| M | 21 | 0 | 0 | 0 | 0 | 0 | 0 |  | Landscape | 200 |  | 11 |
| F | 20 | 1 | 0.5 | 0 | 0.5 | 0.5 | 0 |  | Landscape | 200 |  | 30 |
| M | 21 | 1 | 0.5 | 0 | 1 | 0.5 | 0 |  | Landscape | 200 |  | 12 |
| F | 20 | 1 | 0.5 | 0 | 1 | 0.5 | 0 |  | Landscape | 100 |  | 26 |
| F | 20 | 1 | 0.5 | 0 | 1 | 0 | 0 |  | Fearful face | 200 |  | 16 |
| M | 21 | 1 | 0 | 0 | 1 | 0 | 0 |  | Fearful face | 500 |  | 19 |
| F | 22 | 0 | 0 | 0 | 0 | 0 | 0 |  | Fearful face | 200 |  | 27 |
| F | 23 | 1 | 1 | 0 | 1 | 1 | 0 |  | Landscape | 100 |  | 26 |
| M | 20 | 1 | 1 | 0 | 0 | 1 | 0.5 |  | Fearful face | 200 |  | 14 |
| M | 20 | 1 | 1 | 0 | 1 | 1 | 0 |  | Landscape | 500 |  | 21 |
| M | 19 | 1 | 1 | 0 | 1 | 1 | 0 |  | Landscape | 100 |  | 22 |
| M | 18 | 1 | 1 | 0 | 1 | 0.5 | 0 |  | Landscape | 200 |  | 25 |
| F | 20 | 1 | 1 | 0 | 1 | 0.5 | 0 |  | Landscape | 100 |  | 22 |
| F | 18 | 1 | 1 | 0 | 1 | 0 | 0 |  | Fearful face | 200 |  | 18 |
| M | 20 | 1 | 0.5 | 0.5 | 1 | 1 | 0 |  | Landscape | 500 |  | 17 |
| F | 20 | 1 | 0.5 | 0 | 1 | 0.5 | 0 |  | Fearful face | 200 |  | 9 |
| M | 19 | 1 | 0 | 0 | 0.5 | 0.5 | 0 |  | Fearful face | 500 |  | 19 |
| F | 22 | 1 | 0.5 | 0 | 0 | 0 | 0 |  | Fearful face | 100 |  | 21 |
| M | 19 | 1 | 0.5 | 0 | 1 | 1 | 0 |  | Landscape | 100 |  | 20 |
| F | 19 | 1 | 1 | 0 | 1 | 1 | 0 |  | Fearful face | 100 |  | 2 |
| M | 19 | 0 | 0 | 0 | 0 | 0.5 | 0 |  | Landscape | 200 |  | 14 |
| M | 20 | 1 | 1 | 0 | 1 | 1 | 0 |  | Landscape | 500 |  | 3 |
| M | 20 | 1 | 1 | 0 | 1 | 1 | 0 |  | Landscape | 200 |  | 27 |
| M | 19 | 0 | 0 | 0 | 0 | 0 | 0 |  | Landscape | 200 |  | 21 |
| M | 22 | 1 | 1 | 0 | 1 | 1 | 0 |  | Fearful face | 100 |  | 13 |
| M | 18 | 1 | 0 | 0 | 1 | 1 | 0 |  | Fearful face | 500 |  | 6 |
| M | 18 | 1 | 0.5 | 0 | 1 | 0.5 | 0 |  | Landscape | 200 |  | 23 |
| M | 18 | 1 | 1 | 1 | 1 | 1 | 0.5 |  | Landscape | 200 |  | 10 |
| F | 18 | 1 | 1 | 0 | 1 | 1 | 0 |  | Landscape | 200 |  | 29 |
| F | 18 | 1 | 1 | 0 | 1 | 1 | 0 |  | Landscape | 200 |  | 3 |
| F | 18 | 1 | 0.5 | 0 | 1 | 1 | 0 |  | Landscape | 200 |  | 17 |
| F | 18 | 0 | 0.5 | 0 | 0.5 | 0.5 | 0 |  | Landscape | 500 |  | 4 |
| M | 19 | 1 | 1 | 0 | 1 | 1 | 0 |  | Fearful face | 100 |  | 24 |
| F | 18 | 1 | 0.5 | 0 | 1 | 1 | 0 |  | Landscape | 100 |  | 13 |
| M | 18 | 1 | 1 | 0.5 | 1 | 1 | 1 |  | Fearful face | 500 |  | 30 |
| F | 18 | 1 | 1 | 0 | 1 | 1 | 0 |  | Landscape | 100 |  | 13 |
| M | 19 | 1 | 1 | 0 | 1 | 1 | 0 |  | Fearful face | 100 |  | 26 |
| F | 19 | 1 | 0.5 | 0 | 1 | 0.5 | 0 |  | Landscape | 200 |  | 5 |
| M | 18 | 1 | 1 | 0 | 1 | 1 | 0 |  | Fearful face | 200 |  | 11 |
| F | 18 | 1 | 1 | 0 | 0.5 | 0.5 | 0 |  | Landscape | 500 |  | 19 |
| F | 19 | 0 | 0 | 0 | 0 | 0 | 0 |  | Landscape | 100 |  | 30 |
| F | 19 | 0.5 | 0 | 0 | 0.5 | 0 | 0 |  | Landscape | 100 |  | 12 |
| F | 21 | 1 | 0.5 | 0 | 1 | 0.5 | 0 |  | Landscape | 200 |  | 8 |
| M | 20 | 1 | 0.5 | 0 | 1 | 0 | 0 |  | Fearful face | 100 |  | 15 |
| M | 20 | 1 | 1 | 0 | 1 | 1 | 0 |  | Fearful face | 100 |  | 17 |
| F | 18 | 1 | 1 | 0 | 1 | 1 | 0 |  | Fearful face | 200 |  | 25 |
| F | 19 | 1 | 1 | 0.5 | 1 | 1 | 0.5 |  | Landscape | 200 |  | 22 |
| F | 20 | 0.5 | 1 | 0 | 1 | 1 | 0 |  | Fearful face | 200 |  | 17 |
